# The Interaction Between Leadership, the Patient-to-Nurse Ratio and Nurses’ Work-Life Balance in the Psychiatric Inpatient Setting in Switzerland: A Secondary Data Analysis of Cross-Sectional Data

**DOI:** 10.1007/s10488-022-01239-6

**Published:** 2022-12-15

**Authors:** Evgenia Zraychikova, Beatrice Gehri, Franziska Zúñiga, Stefanie Bachnick, Michael Simon

**Affiliations:** 1 Psychiatric University Hospital Zurich (PUK), Lenggstrasse 31, CH-8032 Zurich, Switzerland; 2 Department Public Health (DPH), Faculty of Medicine, Institute of Nursing Science (INS), University of Basel, Bernoullistrasse 28, CH-4056 Basel, Switzerland; 3 Department of Psychiatry, University of Basel, CH-4002 Basel, Switzerland; 4 Hochschule für Gesundheit, DPW Department of Nursing Sciences, University of Applied Sciences, Gesundheitscampus 6-8, DE-44801 Bochum, Germany

**Keywords:** Psychiatric nurses, Work-life balance, Psychosocial work environment, Leadership, Patient-to-nurse ratio

## Abstract

Psychiatric nurses’ work environment factors, including long hours, heavy workloads and leadership issues, can serve as barriers to achieving a healthy work-life balance. However, for both individuals and organizations, that balance is crucial as it is a key determinant of job satisfaction and leaving intentions. To address the limiting evidence to that topic, this study had two objectives: (1) to describe the work-life balance of nurses working in psychiatric inpatient settings; and (2) to examine those nurses’ work-life balance and its associations with individual (i.e., age, gender), psychosocial (i.e., leadership) and structural factors (i.e., employment percentage). To analyze these factors and their impacts, we conducted a cross-sectional study in a sample of 1209 nurses from 116 units in 13 psychiatric hospitals of the German-speaking part of Switzerland and analyzed the resulting data via multilevel analysis. This led to three main results. First, nurses reported a high mean value regarding their work-life balance. Second, multivariable regression results indicated that their work-life balance ratings correlated directly with certain psychosocial work environment factors (leadership and support of nurses, perceived staffing resources) and inversely with structural factors (employment percentage, overtime). And third, we found an interaction between leadership and support of nurses and the patient-to-nurse ratio: the lower the leadership level, the stronger the inverse association between patient load and work-life balance. No individual factors were significantly associated with work-life balance. Overall, though, we found that organizational factors are vital to nurses’ work-life balance. Therefore, interventions to improve nurses’ work-life balance should be institution-level, and should focus on improving either leadership or structural factors, e.g., employment percentage, overtime, and patient-to-nurse ratios.

## Introduction

Nurses’ working conditions often challenge the attainment of a healthy work-life balance. Commonly-cited issues include shift work, staff shortages, time pressure, and physical strain. However, work-life balance is crucial both for nurses and for the institutions that employ them. For individuals, an imbalance may lead to health problems such as poor sleep quality, stress reactions (behavioral and cognitive stress symptoms), or burnout (Hämmig, 2018; Peter et al., 2020). For organizations and their employees, job dissatisfaction and turnover intentions count as negative consequences of lacking work-life balance (Peter et al., 2020). Especially burnout and job dissatisfaction lead to leaving intentions, increasing the already straining lack of nurses in the labor pool (Schwendimann et al., 2014). Considering that, in comparison to most other health care settings, work in mental health care frequently is connected to a risk of violent acts and other physical and psychological aggression (Schlup et al., 2021), we expected these problems to be particularly acute in psychiatric settings. Also, as recruiting and retaining psychiatric nurses is usually a challenge, it is important to investigate their work-life balance and to understand its influencing factors.

The current study provides new evidence based on a secondary analysis of data from a large-scale survey of nurses on 116 units in 13 psychiatric hospitals in Switzerland’s German-speaking region. In addition to facilitating a robust description of the overall level of work-life balance among nurses in psychiatric settings, the size of this dataset allowed us to identify relevant individual and organizational factors and their associations with work-life balance. Moreover, we employed a novel method of measuring staffing levels (Bachnick et al., 2018). This provided a particularly accurate estimate of patient-to-nurse ratios and their link to work-life balance.

## Background

A desirable work-life balance is defined as “a high level of engagement in work life as well as non-work life with minimal conflict between social roles in work and non-work life” (Sirgy & Lee, 2018, p. 232). Role conflicts can arise from unsuccessful coordination of work and non-work activities (Frone, 2003). Sexton et al. (2017) suggest that work-life balance is intricately connected to various behaviors, such as skipping meals, eating poorly balanced meals, working through a shift without breaks, arriving home late from work, having sleeping difficulties, sleeping less than 5 hours a night, and commonly changing personal or family plans because of work.

**Fig. 1:**
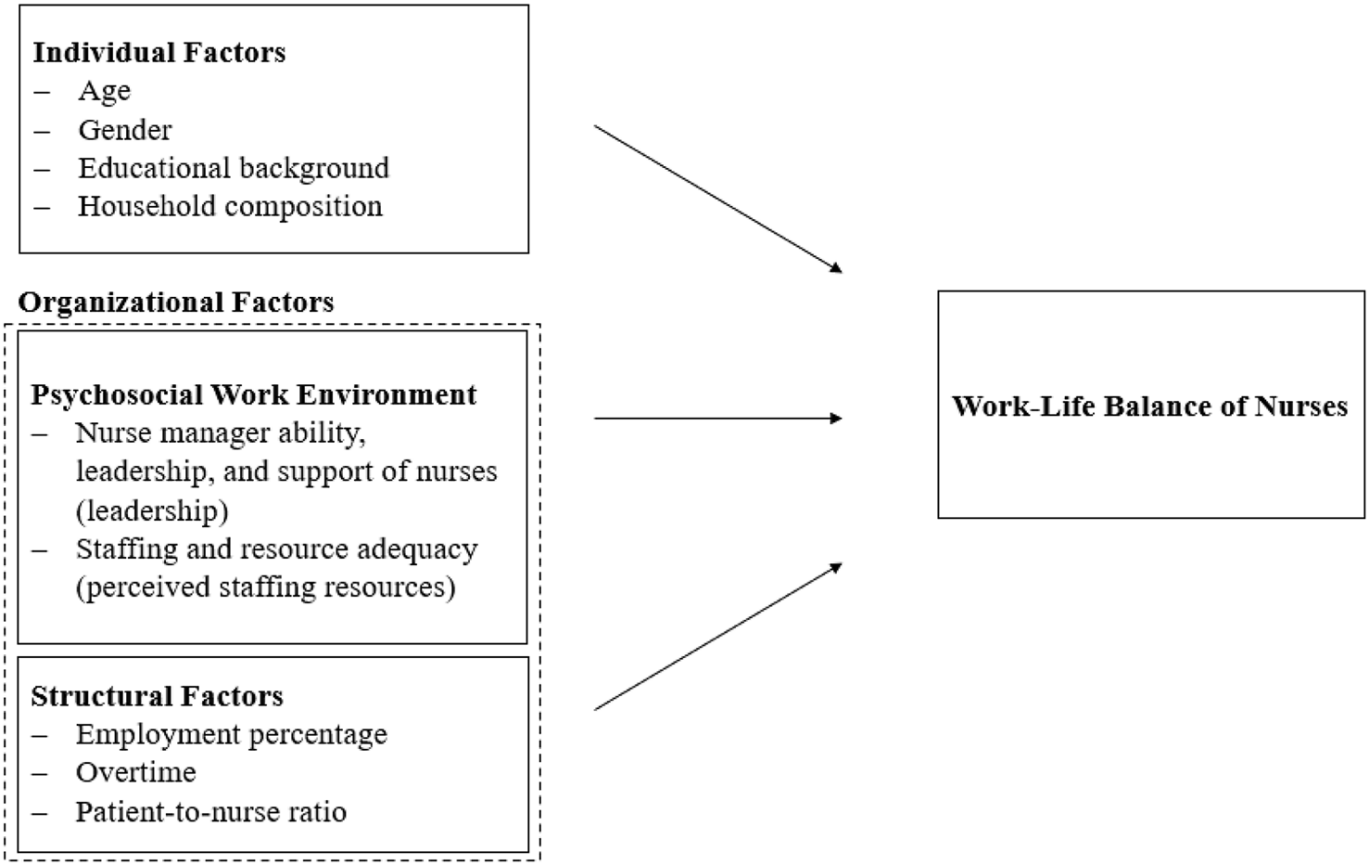
Conceptual model of the influencing factors on nurses’ work-life balance in psychiatric hospitals

Both individual and organizational factors are associated with work-life balance. Based on the literature, work-life balance is explainable via three groups of factors: those specific to individuals, to psychosocial work environments, and to institutional structures (Fig. 1).

*Individual factors* are variables that constitute an individual’s life circumstances and the role demands they face, which in turn influence work-life balance (Dex & Bond, 2005). *Age* is also linked to work-life balance due to changes both in the demands individuals face and their capacities to meet those demands as they age. This is congruent with Dex and Bond’s (2005) finding that work-life balance problems increase with age.

We further hypthesize that *gender* would influence work-life balance due to the unequal distribution of responsibilities at home (Sexton et al., 2017). Recent findings indicate that employed women tend to spend more time on household responsibilities such as childcare and housekeeping than employed men. As a result, they experience more time pressure, leading to problems balancing their work with their private lives (Starmer et al., 2019). Furthermore, *household composition* is likely to play a major role. We assume that the number of children per caretaker influences the household’s overall demands and responsibilities: the larger the household, the greater the barrier to balance.

Finally, we expected that *educational background* also would influence work-life balance. As registered nurses have more education than licensed practical nurses, we reasoned that they should demonstrate higher overall problem-solving competencies, particularly regarding complex patient needs (Nurumal et al., 2017).

Regarding *psychosocial work environment factors*, on the one hand, leadership strongly influences how employees perceive and experience their organization’s work environment. Unit leaders moderate between structural requirements and resources on the one side and staff needs on the other. Yildirim and Aycan (2008) noted that more instrumental and emotional support from a supervisor correlates significantly with lower work/family conflict and higher job satisfaction. Cummings et al. (2018) stated that certain leadership styles promote a more comfortable organizational climate, thereby reducing emotional exhaustion and job stress. Therefore, we expected that the perception of leadership, encompassing nurse manager ability, leadership and support of nurses, would correlate directly with work-life balance.

Moreover, nurses’ perceptions of staffing and resource adequacy might play an important role for work-life balance. Perceived workload has emerged as an important determinant of work-life balance in the health-care setting in previous research (Holland et al., 2019).

The third and final group, *structural factors*, are the organizational characteristics that color nurses’ work experience. For example, by increasing the time nurses spend at work, *working overtime* leaves correspondingly less time to engage in personal activities. As a result, it has been associated with decreases in work-life balance (Lobo et al., 2017; Watanabe & Yamauchi, 2016). Similarly, by indicating how much time employees spend at work, *employment level* also decides the maximum amount of time left to fulfill private plans and obligations. Therefore, it is likely to influence the work-life balance (Beham et al., 2012; Hämmig et al., 2009).

Finally, the *patient-to-nurse ratio* must be considered. According to Hämmig’s 2018 systematic review, increases in the patient-to-nurse ratio are associated with increases in emotional exhaustion, an outcome of an unbalanced relationship between work and private life (Hämmig, 2018; Wynendaele et al., 2019).

In the psychiatric setting, only two studies concerning work-life balance have been carried out to date. In Pryce et al. (2006) intervention study (in a psychiatric hospital in Denmark), nurses designed their own work-rest schedules with an open-rotation scheduling system. After the intervention, a significant increase of work-life balance in psychiatric nurses was observed. Another study in the psychiatric setting was conducted with 180 nurses in Japan employing a cross-sectional design. According to the results, work-family conflict mediates the relationship between occupational factors and burnout (Sugawara et al., 2017).

Despite the value of these findings, more evidence clearly is needed on work-life balance and its factors concerning nurses in psychiatric settings. Therefore, this study’s goals are twofold: (a) to describe the work-life balance of nurses in a psychiatric inpatient setting in Switzerland and (b) to investigate associations between that balance and individual, psychosocial, and structural factors in those nurses’ psychiatric inpatient setting.

## Methods

### Design

We conducted a secondary data analysis of the ongoing multi-center cross-sectional “Matching registered nurse services with changing care demands in Psychiatry Hospitals” (Match^RN^ Psychiatry) Project (Gehri et al., 2021). Match^RN^ Psychiatry assesses the quality of care in psychiatric hospitals considering structures, processes, patient, and nurse outcomes.

### Setting and Sample

Overall, Switzerland has 50 psychiatric hospitals. Their median size is 76 beds (Bundesamt für Statistik [BFS], 2013, 2019). The most frequent diagnosis groups in inpatient psychiatric hospitals are affective disorders (32.7%), mental and behavioral disorders associated with psychotropic substance use (19.4%), schizophrenia (16.9%), and neurotic, stress and somatoform disorders (13.2%) (Schuler et al., 2019). The mean length of stay is 34.4 days (BFS, 2021).

We invited 40 psychiatric hospitals—members of the Swiss Psychiatric Nursing Leaders Association—to participate in the study. Thirteen hospitals from the German-speaking part of Switzerland consent in writing and were included in the study. Within the participating hospitals’ 116 adult inpatient care units (e.g. acute care, gerontological care, psychotherapeutic care), registered nurses (RN) and licensed practical nurses were surveyed using self-administered questionnaires. Nursing students and units focusing on forensic psychiatry were excluded. Further details regarding the Match^RN^ Psychiatry study design are reported elsewhere (Gehri et al., 2021). The sample used for this sub-study consisted of 1209 nurses from 116 units in 13 psychiatric hospitals. The mean response rate across all hospitals was 71.5% (range: 58.1–88.1%). The majority (70.3%) of participants were female; most (89.1%) were registered nurses. Detailed information on the characteristics of the studied variables are presented in Table 1.

**Table 1:** Descriptive statistics, work-life balance and predictors

	Valid *n * (%)	Missing (%)	Mean (± SD)
Outcome variable			
Work-life balance (range: 1–4)	1189	1.7%	3.1 (0.5)
Independent variables			
Gender	1158	4.2%	
Female	814 (70.3)		
Male	344 (29.7)		
Age (years) (range: 17–67)	1144	5.4%	40.2 (12.7)
≤30	346 (30.2)		
31–40	272 (23.8)		
41–50	210 (18.4)		
≥51	316 (27.6)		
Educational background	1161	4.0%	
Registered nurse	1034 (89.1)		
Licensed practical nurse	127 (10.9)		
Employment percentage (%) (range: 10–100)	1142	5.5%	83.5 (19)
≤50	111 (9.7)		
51–80	397 (34.8)		
81–100	634 (55.5)		
Household composition	1143	5.5%	
Alone	236 (20.6)		
With another adult	544 (47.6)		
With children	56 (4.9)		
With another adult and children	307 (26.9)		
Leadership (range: 1–4)	1191		3.1 (0.7)
Perceived staffing resources (range 1–4)	1191		2.8 (0.7)
Unit level variables (n = 116 units)			
Overtime (minutes)			14.5 (10.9)
Patient-to-nurse ratio			
Early shift			7.5
Late shift			9.4
Night shift			16.2

### Data Collection

Nurse-level data were collected between September 2019 and March 2020. Participating hospitals could choose between online (unipark) or paper-and-pencil questionnaire surveys. At each participating hospital, one contact person was responsible for the organization and distribution of the questionnaire. Participation in the study was voluntary. Participants implied their informed consent by completing and submitting the questionnaire.

### Variables and Measurements

The 164-item Match^RN^ psychiatric nurse survey is based on the Match^RN^ questionnaire (Bachnick et al., 2018). It contains questions on structural factors, safety culture, the quality of the nurse work environment, and work processes, as well one set of items on the respondent’s socio-demographic characteristics and another on nurse outcomes including job satisfaction and wellbeing (Kristensen et al., 2005).

### Outcome Variable

To measure work-life balance, we used the previously-validated work-life climate scale (Sexton et al., 2017). This scale’s core consists of eight items, each asking the incidence over the past week of a behavior related to work-life balance: skipped a meal, ate a poorly balanced meal, worked through a day/shift without breaks, arrived home late from work, had sleeping difficulties, slept less than 5 hours in a night, changed personal/ family plans because of work, and felt frustrated by technology. The items are measured on 4-point Likert-type scales, with the following response options: “all of the time (5–7 days) (1 point);” “occasionally or a moderate number of times (3–4 days) (2 points);” “some or a little of the time (1–2 days) (3 points);” and “rarely or none of the time (less than 1 day) (4 points).” A higher score indicates better work-life balance. Cronbach’s α was 0.74 in this study. For our analysis, we used the mean of all items as a metric variable (range: 1–4).

### Independent Variables

*Individual factors*: To describe individual factors’ associations with work-life balance, we measured four socio-demographic characteristics: age (in years), gender (male = 0; female = 1), household composition (alone = 1/with one adult = 2/with child or children = 3/with an adult and child or children = 4), and educational background (RN = 0; licensed practical nurse = 1). RNs require either a bachelor’s degree or an advanced federal diploma (3-to 6-year education), licensed practical nurses require training and a federal diploma of vocational education (3-year education) (see Fig. 1 for an overview of all covariates of work-life balance).

*Psychosocial work environment*: To measure “Nurse Manager Ability, Leadership, and Support of Care Workers” (leadership) and “Staffing and Resource Adequacy” we used two subscales of the revised Practice Environment Scale–Nursing Work Index (PES-NWI) (Lake, 2002). Items were rated on a 4-point Likert-type scale (from 1 “strongly disagree” to 4 “strongly agree”). Leadership was assessed with four items, including statements on the extent to which the direct supervisor was supportive of the care workers on their team, or the extent to which they considered their nurse manager a good manager and leader.

Staffing and resource adequacy was measured with four items, e.g., the extent to which enough time was provided to discuss patient care problems or to which the enough support personnel were available to complete all necessary tasks. In this study Cronbach’s α was 0.82 for leadership and 0.74 for staffing and resource adequacy. For the analysis, the mean of all items per subscale was calculated.

*Structural factors*: As structural factors, we included employment percentage, overtime and patient-to-nurse ratio. Employment percentage was measured on a numeric scale at the individua level (10 to 100%). Overtime was measured in minutes per shift, aggregated to the unit mean.

Based on Bachnick et al. (2018) approach, we calculated nurse staffing level as the ratio of the total number of patients to the total number of registered nurses (patient-to- registered-nurse ratio) on the unit during the most recent shift. We used a mixed effect model and adjusted the nurse staffing level for three variables: (a) shift (morning, afternoon, or night); (b) the number of patients for whom each nurse was directly responsible per shift (adjusted for the number of patients admitted or discharged); and (c) skill mix (calculated as the number of registered nurses divided by the combined number of registered nurses and licensed practical nurses). As a standardized staffing measure, we used the empirical Bayes estimate obtained via the random effect model.

### Data Analysis

First, descriptive statistics (frequencies, percentages, means, standard deviations) were calculated to describe all studied variables. Second, to explore the relationships linking individual factors, psychosocial work environment, and structural factors with work-life balance, we used a two-level generalized linear mixed model (GLMM). Overtime and patient-to-nurse ratio represent unit-level constructs (level 2). Multilevel analysis was indicated based on the intraclass correlation 1 (ICC1). For work-life balance this was 0.12 on the unit level and 0.04 on the hospital level (Biemann et al., 2012; LeBreton & Senter, 2008). According to the literature, where ICC1 values exceed 0.05, the data’s hierarchical structure should be considered (Snijders & Bosker, 2011).

First, we computed separate bivariate regressions for work-life balance and every independent variable. Second, we computed a regression model that included all independent variables. This model showed that the individual factors were non-significant (*p* > 0.05). To arrive at a parsimonious model, then, these variables were excluded from further analysis. For the first model presented in our Results section (model 1, Table 2), we regressed work-life balance against the psychosocial work environment and structural factors. In a final model, we extended this model via an interaction term between leadership and patient-to-nurse ratio, i.e., an consistent effect produced by combinations of these two factors. To compare the models’ relative fits, we used Akaike’s information criterion (AIC). Lower AIC values indicate a better fit (Gelman & Hill, 2006). Listwise deletion was applied to handle missing values. Data analyses were performed using R version 3.6.1. (Team, 2013) and R package lme4 (Bates et al., 2011).

**Table 2:** Associations of work-life balance with predictors

	Bivariate		Model 1		Model 2	
	coef (t)		coef (t)		coef (t)	
Age	0.001 (0.663)					
Male^1^	0.003 (0.104)					
Educational background^2^	0.021 (0.466)					
Employment percentage	− 0.002 (− 2.166)	*	− 0.002 (− 2.82)	**	− 0.002 (− 2.745)	**
HH with another adult^3^	0.041 (1.062)					
HH with children	− 0.009 (− 0.118)					
HH with another adult and children	0.046 (1.085)					
Leadership	0.258 (12.160)	***	0.132 (5.256)	***	0.136 (5.409)	***
Perceived staffing resources	0.330 (16.160)	***	0.244 (9.498)	***	0.242 (9.433)	***
Patient-to-nurse ratio	− 0.024 (− 0.647)		− 0.036 (− 1.376)		− 0.220 (-2.027)	*
Overtime	− 0.010 (− 5.43)	***	− 0.005 (− 3.49)	***	− 0.006 (− 3.615)	***
Leadership x patient-to-nurse ratio					0.0597 (1.749)	✝
*n*			1138		1138	
AIC			1414.417		1418.281	

Reference categories: ^1^ female, ^2^ registered nurses, ^3^ HH alone

✝ p < 0.1, *p < 0.05, **p < 0.01, ***p < 0.001

*HH* Household composition

## Results

### Descriptive Results

The mean *work-life balance* rating was 3.1 (SD: 0.5), corresponding roughly to the “some or a little of the time (1–2 days)” category. This indicates a rather high work-life balance. The work environment ratings were high for *leadership*, with an average rating of 3.1 (SD: 0.7). This is slightly above “rather agree” (scale range: 1–4). With an average rating of 2.8 (SD: 0.7) (scale range 1–4), *staffing and resource adequacy* was rated just below “rather agree.”

On the unit level on average, one nurse was responsible for 7.5 patients during the early shift, 9.4 during the late shift, and 16.2 during the night shift. Nurses reported an average of 15 min of overtime for their most recent shift.

### Factors Related to Work-Life Balance

We found that work-life balance correlated significantly with employment percentage, the two work environment factors (leadership and resource adequacy) and overtime (Table 2). Employment percentage and overtime correlated inversely with work-life balance, i.e., increases either in employment percentage or in overtime led to lower work-life balance. Regarding work environment factors, increases in *leadership* or in *staffing and resource adequacy* were associated with higher work-life balance. None of the four analysed demographic characteristics—*gender*, *age*, *educational background*, or *household composition*—or *patient-to-nurse ratio* were associated significantly with work-life balance.

With our second model (model 2, Table 2), we tested for an interaction between *leadership* and *patient-to-nurse ratio*. In this case, most results were the same as in model 1. However, whereas no significant correlation was previously indicated between *patient-to-nurse ratio* and *work-life balance*, this model yielded an inverse interaction between *leadership* and *patient-to-nurse ratio*—just above the threshold for significance (p = 0.081). Moreover, the combination of decreased management effectiveness and increased patient-to-nurse ratios significantly affected work-life balance.

In fact, with highly-rated leadership, the association between the patient-to-nurse ratio and work-life balance was close to zero. If nurses’ leadership ratings were lower, though, the association of even a fairly small increase in the patient-to-nurse ratio was associated with lower work-life balance.

For example, with a good leadership rating of 3.74, a unit increase in the patient-to-nurse ratio (i.e. one patient more per nurse) would leave the work-life balance score practically unchanged with a slight, increase of 0.003 scale points. If that leadership rating is low, i.e. 2.44, though, the same increase in patient-to-nurse ratio is associated with 0.074 less scale points in work-life balance. To summarize this finding, our results indicate that the theoretically expected negative effect of increasing workload only holds in cases of low-rated leadership.

## Discussion

The current study provides valuable insights into the work-life balance of nurses in psychiatric hospitals in the German-speaking part of Switzerland, while clarifing associations between work-life balance and factors relating to individual nurses, their psychosocial nurse work environment, and their institutions’ structural details.

First of all, our sample’s nurses reported high mean values regarding work-life balance. On average, they only reported performing behaviors related to a poor work-life balance some or a little of the time. This result is comparable to those of Schwartz et al. (2019) survey analysis, for which more than 60% of participating acute-care nurses rated their work-life balance as good or higher.

Our second key finding is that work-life balance is related exclusively to psychosocial work environment and structural factors. More concretely, we found statistically significant associations between work-life balance and leadership, staffing and resource adequacy, employment percentage, overtime, and patient-to-nurse ratio. In contrast to other studies, we did not find a significant correlation of work-life balance with individual factors including age, gender, educational background, or household composition (Dex & Bond, 2005; Sexton et al., 2017; Starmer et al., 2019). Overall, these findings underscore the relevance of organizational factors to work-life balance.

Our positive association between leadership and work-life balance is consistent with Schwartz et al. (2019) findings. This link might be explained by supervisory support and the organizational culture. First, previous research found that, for nurses, positive perceptions of their leadership is linked to decreased work-related stress (Cummings et al., 2018). Arguably, unit leaders who skillfully navigate between patient requirements and available resources, while showing concern for staff and patient needs, are likely to minimize stress levels, thereby improving their team members’ work-life balance. Second, our findings are consistent with those of Sexton et al. (2017), who linked work-life balance to workplace culture.

Behaviors related to work-life balance, such as taking breaks, maintaining a balanced diet, and going home on time are all likely to be encouraged in a team with a favorable workplace culture. Previous research has shown that certain leadership styles are connected to the organizational culture (Cummings et al., 2018). Under a manager who shows a commitment to behaviors conducive to a work-life balance, then, we would expect more healthcare workers to adopt those behaviors. While we measured nurses’ perceptions of leadership and not the actual leadership style, it is reasonable to assume that unit leaders who encourage a favorable working culture also are perceived as more supportive.

Similarly, the association between work-life balance and nurse-perceived staffing and resource adequacy can be explained by supervisory support. Cummings et al. (2018) found that, where leaders were supportive and/or authentic, staffing was perceived as better or received higher ratings. Similarly, it is reasonable to assume that perceived adequacy of staffing resources leads to better quality of care, less stress on the nurses and more job satisfaction among staff members. In turn, all of these benefits favor work-life balance.

The inverse association between work-life balance and both employment percentage and overtime could be explainable in terms of conflicts between private and work life, particularly a lack of time to fulfill private obligations. First, nurses with higher employment percentage are exposed more often and more pervasively to work-related stress. Hospital work is stressful. Therefore, full-time nurses are more exhausted and have less time to regenerate between shifts. Second, due to the lack of time, these nurses have less time to attend to their private matters.

In contrast, part-time employees have more possibilities to manage the conflicting demands of work and private life. The resulting flexibility would favor a higher work-life balance. This explanation is consistent with Hämmig et al. (2009) clear finding that employment level and regular overtime are associated with higher levels of work-life conflict (see also Beham et al., 2012). Our findings are also congruent with those of Watanabe and Yamauchi (2016), who found that involuntary overtime work had a strong negative effect on work-life balance.

Although the observed interaction was relatively weak, another key finding was the interaction between (poorly-perceived) leadership and the patient-to-nurse ratio. The more favorable the participants’ ratings of their supervisors’ leadership, the weaker the correlation between higher patient-to-nurse ratios and work-life balance. That is, where leadership received high ratings, no significant association existed between patient-to-nurse ratio and work-life balance. However, where the respondents perceived poor leadership, higher nurse-to-patient ratios were linked to poorer work-life balance. One interpretation of this result is that high-level leadership skills have a buffering effect on poor staffing levels. This could reflect higher-rated leaders’ greater ability to manage available resources or to motivate their teams despite resource deficits. In contrast, low-rated leaders likely respond to similar shortfalls in ways that increase their teams’ work-associated stress, thereby decreasing work-life balance. This finding requires confirmation in future research.

On the theoretical level, by emphasizing effective leadership’s mitigating effect on how organizational gaps impact work-life balance, it shows the close link between the psychosocial work environment and how leadership can partially compensate for lack of structural ressources such as the patient-to-nurse ratio. In practical terms, especially considering the increasing difficulty of finding and keeping skilled nurses, the evidence presented above supports the view that investment in leadership training (i.e. well-prepared supervisors) is an important means to counteract the negative impacts of poor staffing levels on work-life balance. However, it does not follow that investments in leadership training alone are sufficient for the sustainable functioning of the organization. At least in the midterm, investments in the general staffing level are likely to be necessary as well.

## Strengths and Limitations

This study has several important strengths. For example, regarding psychiatric hospitals in the German-speaking part of Switzerland, it reports on the first investigation of individual and organizational factors’ associations with their nurses’ work-life balance. Moreover, the study sample (n = 1209 nurses) was sufficiently large to estimate robust effects. Finally, to estimate our patient-to-nurse ratios, we used a novel measure—one that accounts for shift, skill-mix, and number of patients (Bachnick et al., 2018).

However, our study also has notable limitations. First, its cross-sectional design allows no inferences regarding causality, including even its direction between variables. Further research, applying a longitudinal study design and experimental methods, will be necessary to investigate the impacts of the psychosocial work environment, structural and individual factors on work-life balance. Second, common method bias due to the use of self-reported measures might have influenced the results. Last, as data collection was confined to the German-speaking part of Switzerland, it is not possible to generalize the results to Switzerland’s French- and Italian-speaking regions or to Germany and Austria.

## Conclusion and Implications for Practice

Our results indicate that the studied nurses’ work-life balance is strongly associated both with structural factors of their hospitals and with their psychosocial work environment. Regarding the latter, the interaction between quality of leadership and patient-to-nurse ratio, although weak, plays a role regarding nurses’ work-life balance that requires further study. This suggests that unit leaders should promote a collaborative work environment based on an organizational culture of support, encouraging behaviors that support work-life balance in everyday work, e.g., taking breaks and going home on time. Furthermore, unit leaders should recognize their role modeling function and practice work-life balancing behaviors. In addition, organizations can improve their workplace culture by providing programs to train unit leaders in authentic, supportive, and transformational leadership styles, i.e., those shown to improve key nurse outcomes (Cummings et al., 2018). Relatively small investments in leadership quality promise high-value returns regarding nurses’ work-life balance.

Regarding influential structural factors, we found that nurses who have to spend more hours on the job—particularly as non-voluntary overtime—clearly have a worse work-life balance than those who work regular or even reduced hours. Thus, in addition to minimizing or avoiding the need for nurses to work overtime, e.g., via re-delegation of tasks, organizations and leaders are advised to establish transparent regulations regarding how it is assigned and remunerate staff fairly when they stay late. Second, considering both that even normal nursing workloads are quite heavy and that structural factors dictate standard employment conditions, one possible way to improve nurses’ work-life balance would be to establish a maximum working week of 4 days (33.6 h). In addition to facilitating a healthy work-life balance, this would make the nursing profession more attractive, thereby helping counteract the current shortage of nurses. However, either improving the situation regarding nurses’ overtime or reducing the standard number of hours they work per week might require a combination of political action and enforcement of the new regulations.

Our finding regarding the interaction between patient-to-nurse ratio and leadership also bears an important implication for practice: by encouraging and investing in beneficial leadership skills, organizations can compensate to some extent for fluctuations in care demand or staff. That is, skilled leaders increase organizations’ flexibility, allowing them to respond effectively to opportunities and constraints as they arise. For example, without changes to leadership style, the simplest way organizations can improve work-life balance is simply by recruiting additional nurses. If this is not feasible, they could foster beneficial and easily- implemented leadership practices such as encouraging regular breaks or calling team meetings. A team meeting, representing an opportunity to give support to team members.
